# Drug-Drug Interaction Potentials of Tyrosine Kinase Inhibitors via Inhibition of UDP-Glucuronosyltransferases

**DOI:** 10.1038/srep17778

**Published:** 2015-12-08

**Authors:** Nan Zhang, Yong Liu, Hyunyoung Jeong

**Affiliations:** 1 Department of Pharmacy Practice, College of Pharmacy, University of Illinois at Chicago, Chicago, IL 60612, United States; 2 Department of Biopharmaceutical Sciences, College of Pharmacy, University of Illinois at Chicago, Chicago, IL 60612, United States; 3 Department of Medicinal Chemistry and Pharmacognosy, College of Pharmacy, University of Illinois at Chicago, Chicago, IL 60612, United States; 4 School of Life Science and Medicine, Dalian University of Technology, Panjin, LN 124221, China

## Abstract

Tyrosine kinase inhibitors (TKIs) are anticancer drugs that may be co-administered with other drugs. The aims of this study are to investigate the inhibitory effects of TKIs on UDP-glucuronosyltransferase (UGT) activities, and to quantitatively evaluate their potential to cause drug-drug interactions (DDIs). Inhibition kinetic profiles of a panel of UGT enzymes (UGT1A1, 1A3, 1A4, 1A6, 1A7, 1A8, 1A9, 1A10, 2B4, 2B7, 2B15, and 2B17) by four TKIs (axitinib, imatinib, lapatinib and vandetanib) were characterized by using hepatic microsomes and recombinant proteins. Lapatinib exhibited potent competitive inhibition against UGT1A1 activity with a Ki of 0.5 μM. Imatinib was found to exhibit broad inhibition on several UGTs, particularly potent competitive inhibition against UGT2B17 with a Ki of 0.4 μM. The TKIs also exerted intermediate inhibition against several UGTs (i.e., UGT1A7 by lapatinib; UGT1A1 by imatinib; UGT1A4, 1A7 and 1A9 by axitinib; and UGT1A9 by vandetanib). Results from modeling for the quantitative prediction of DDI risk indicated that the coadministration of lapatinib or imatinib at clinical doses could result in a significant increase in AUC of drugs primarily cleared by UGT1A1 or 2B17. Lapatinib and imatinib may cause clinically significant DDIs when co-administered UGT1A1 or 2B17 substrates.

Tyrosine-kinase inhibitors (TKIs) are anticancer drugs. Tyrosine kinases phosphorylate the tyrosine residues of proteins involved in the activation of signal transduction cascades that play key roles in biological processes including growth, differentiation and apoptosis in cancer cells^1^. Currently, more than 20 FDA-approved TKIs are used clinically. More than 80% of cancer cases are developed in patients older than 60 years old^2^ who typically have other medical conditions that require drug treatment^3^. As a result, TKIs have been commonly combined with other drugs in cancer patients^4^,^5^, and drug-drug interaction (DDI) involving TKIs is a potential clinical concern.

UDP-glucuronosyltransferases (UGT), a class of phase II enzymes, catalyze the conjugation of glucuronic acid to endogenous substances and exogenous compounds. UGT-catalyzed glucuronidation reactions account for approximately 35% of drugs eliminated by phase II enzymes (or one-seventh of the drugs prescribed in the United States in 2002)^6^. The human UGT superfamily involved in xenobiotics metabolism is comprised of 2 families: UGT1 and UGT2^7^. UGT1A1, 1A3, 1A4, 1A6, 1A9, 2B7 and 2B15 are the main UGTs responsible for drug metabolism^8^ while UGT1A7, 1A8, 1A10 and 2B4 have also been found to metabolize drugs including mycophenolic acid and troglitazone^9^. Most UGT isoforms are expressed in liver except UGT1A7, 1A8 and 1A10 that are expressed mainly in intestines^10^,^11^.

Previous *in vitro* and *in vivo* studies indicate that TKIs may alter the hepatic elimination of co-administered drugs by inhibiting their metabolism. For example, nilotinib and erlotinib inhibit UGT1A1 activity, and gefitinib inhibits UGT1A1, 1A7, 1A9 and 2B7 activities^12^,^13^,^14^,^15^. A clinical study also showed that co-administration of lapatinib with irinotecan led to a ~40% increase in the AUC of SN-38 (an active metabolite of irinotecan and a UGT1A1 substrate)^16^, suggesting the possible inhibition of UGT1A1 activity by lapatinib. However, whether these TKIs affect activities of others UGT isoforms and whether other TKIs affect UGTs remain unknown.

In this study, four commonly used TKIs−axitinib, imatinib, lapatinib and vandetanib (Fig. 1)−were evaluated for their capabilities to inhibit UGT activities. The inhibition kinetics of each compound was further characterized, and the risks for clinically significant drug-drug interactions were estimated.

## Results

### Inhibition of UGT Activity by TKIs

As a preliminary study, we first examined whether TKIs inhibit different UGTs. To this end, axitinib, imatinib, lapatinib, or vandetanib (or vehicle control) was incubated with a UGT substrate (4-methylumbelliferone (4-MU) for all UGTs except for UGT1A4; trifluoperazine (TFP) was used for UGT1A4) and one of recombinant UGT enzymes (UGT1A1, 1A3, 1A6, 1A7, 1A8, 1A9, 1A10, 2B4, 2B7, 2B15, and 2B17). Then, the extent of glucuronide metabolite production was examined. The results showed that at 100 μM concentration, TKIs inhibited the activity of UGT isoforms to varying extent (Table 1). For UGT isoforms whose activity is inhibited by >50% by individual TKIs, IC_50_ values of TKIs were further estimated. The summary of IC_50_ values is shown in Table 2.

### UGT Inhibition Kinetics of TKIs

Except axitinib whose maximum plasma concentration (C_max_) is in the sub-micromolar range^17^, the C_max_ values of the remaining TKIs tested in this study are at the micromolar level at typical oral doses. For TKIs that exhibit IC_50_ values lower or similar to the reported plasma C_max_ (i.e., imatinib against UGT1A1 and 2B17; lapatinib against UGT1A1, 1A4, and 1A7; and vandetinib against UGT1A9), their UGT inhibitory kinetics profiles were further characterized.

**Figure 2:**
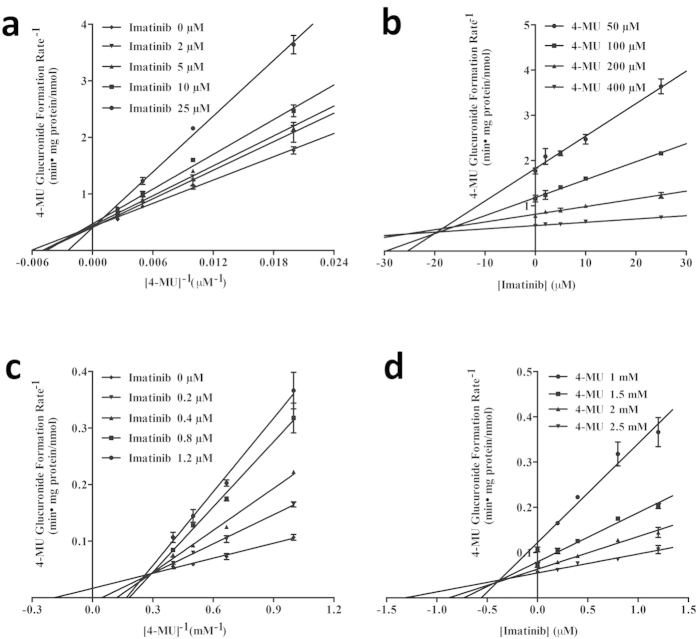
Lineweaver-Burk and Dixon plots for imatinib inhibition of 4-MU glucuronidation by UGT1A1 (**a** and **b**) and UGT2B17 (**c** and **d**) proteins.Data shown are the mean ± standard deviation of triplicate experiment.

**Figure 3:**
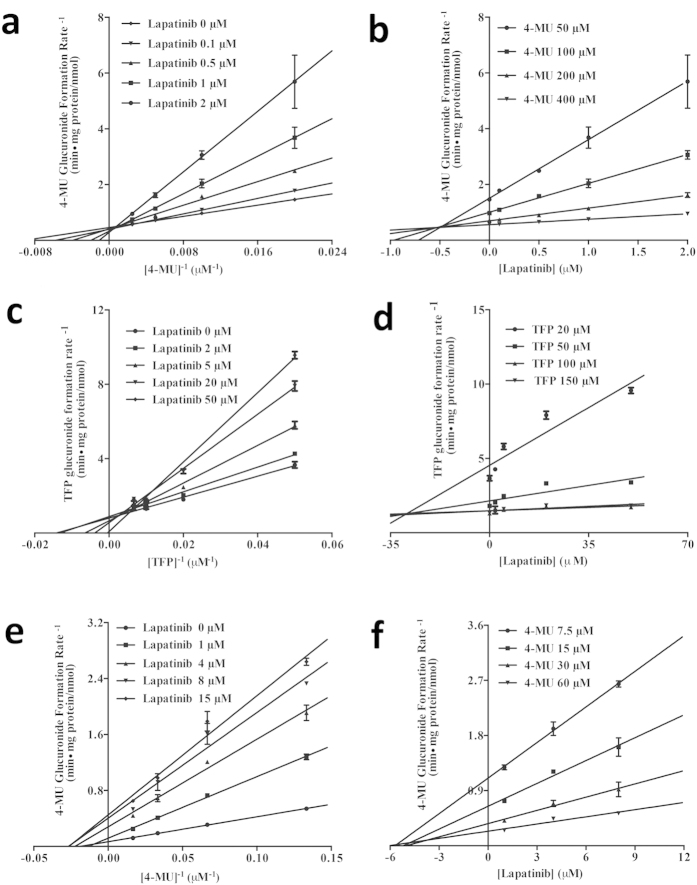
Lineweaver-Burk and Dixon plots for lapatinib inhibition of 4-MU (for UGT1A1, (**a**) and (**b**); and UGT1A7, (**e**) and (**f**)) or TFP (for UGT1A4, (**c**) and (**d**)) glucuronidation. Data shown are the mean ± standard deviation of triplicate experiment.

**Figure 4:**
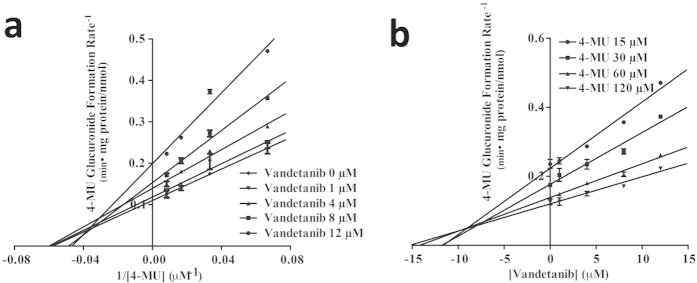
Lineweaver-Burk (**a**) and Dixon plot (**b**) for vandetinib inhibition of 4-MU glucuronidation by UGT1A9 proteins. Data shown are the mean ± standard deviation of triplicate experiment.

The kinetic results were shown in Table 3. Imatinib inhibited UGT1A1 and 2B17 activities (Fig. 2) by a competitive inhibitory mechanism. *K*_i_ values of imatinib were estimated to be 19 μM and 0.4 μM against the UGT1A1 and UGT2B17, respectively. Lapatinib (Fig. 3) exhibited a potent competitive inhibition against UGT1A1 with a *K*_i_ of 0.5 μM, a mixed inhibition against UGT1A4 with a *K*_i_ of 36.4 μM, a non-competitive inhibition with a *K*_i_ of 4.5 μM against UGT1A7. Vandetanib showed a mixed inhibition against UGT1A9 with a *K*_i_ of 9.0 μM (Fig. 4).

### Inhibition SN-38 Glucuronidation by Lapatinib

**Figure 5:**
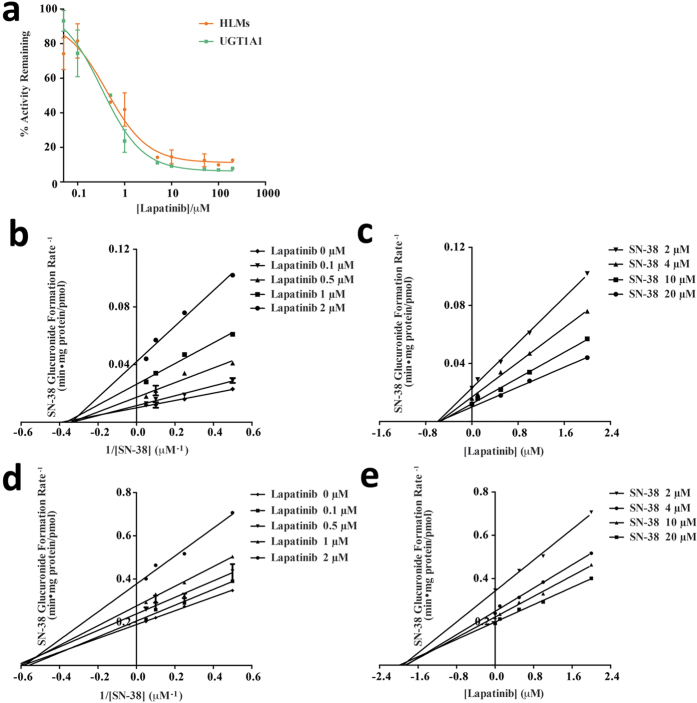
(**a**) Inhibition of SN-38 glucuronidation by lapatinib in human liver microsomes (HLM) or by recombinant UGT1A1 proteins. (**b–e**) Lineweaver-Burk and Dixon plots for lapatinib inhibition of SN-38 glucuronidation by UGT1A1 (**b,c**) or in human microsomes (**d,e**). Data shown are the mean ± standard deviation of triplicate experiment.

Because UGT1A1 inhibition can exhibit substrate-dependency^18^, the inhibition of UGT1A1 activity by lapatinib was further examined using SN-38, a drug mainly metabolized by UGT1A1^19^. IC_50_ values were estimated for the inhibition of SN-38 glucuronidation by lapatinib in human microsomes and recombinant UGT1A1 proteins, and their kinetic parameters were estimated. Lapatinib showed strong inhibition against SN-38 glucuronidation, the IC_50_ of which was 0.33 μM in UGT1A1 and 0.42 μM in human liver microsomes (HLMs) (Fig. 5a). The estimated *K*_i_ values were 0.6 μM and 1.6 μM for UGT1A1 and HLMs, respectively, which were similar to the *K*_i_ values for the inhibition of 4-MU glucuronidation by lapatinib. Interestingly, however, the kinetic profile of inhibition of SN-38 glucuronidation was different from that of 4-MU glucuronidation; while inhibition of 4-MU glucuronidation by lapatinib was competitive, the inhibition of SN-38 glucuronidation by lapatinib (Fig. 5b–e) was non-competitive in both UGT1A1 and HLMs (Table 3).

### Quantitative Prediction of DDI Potential

The risk of DDIs due to the inhibition of UGTs by TKIs was evaluated by predicting the ratio of area under the plasma drug concentration versus time curve (AUC) of a victim drug in the presence and absence of the TKI (i.e., AUC_i_/AUC). To this end, the hepatic or systemic concentrations of each TKI were first estimated using previously reported clinical pharmacokinetic data (Table 4). For the inhibition of UGT1A1, 1A9, and 2B17, the maximum unbound hepatic input concentrations of TKIs were estimated. For the inhibition of UGT1A7, which is mainly expressed in intestines, systemic drug concentration was estimated. The AUC ratios of each inhibitor were estimated based on K_i_ values obtained in this study and the calculated hepatic or plasma concentrations of TKIs. The AUC changes of the UGT2B17 substrate by imatinib (at the time for C_max_) were predicted to be significant. For example, UGT2B17 inhibition by imatinib was predicted to increase the AUC of 4-MU by 140% when the fraction of the UGT substrate metabolized by the inhibited enzyme (*f*_m_) of a victim drug was 90%, which was much greater than the cut-off value for potential clinical significance (i.e., 25%)^20^. Also, lapatinib was predicted to cause ~25% increase in AUC of UGT1A1 substrates (4-MU and SN-38) when the *f*_m_ is 1, indicating a potential DDI risk with clinical significance. On the other hand, axitinib could not cause the increase in AUC of UGT1A4, 1A7, or 1A9 substrates when the *f*_m_ is 1, and the AUC_i_/AUC ratios were close to 1. UGT1A9 inhibition by Vandetanib, UGT1A1 inhibition by imatinib, and UGT1A7 inhibition by lapatinib were predicted to increase the AUC of substrate by about 2%, 3%, and 1%, indicating minor clinical impacts of the TKIs on the pharmacokinetics of other UGT substrates.

## Discussion

In recent years, a number of TKIs of different classes have been approved for cancer treatment. Among these TKIs, several TKIs of the 4-anilinoquinazoline class (erlotinib and gefitinib) and pyridine class (sorafenib) have been shown to inhibit UGTs^12^,^13^,^15^,^21^. In the present study, we selected four commonly used TKIs, including two 4-anilinoquinazolines (lapatinib and vandetanib), one pyrimidine (imatinib) and one indazol (axitinib) to investigate whether these TKIs could inhibit a panel of UGTs activities. To this end, 4-MU was used as a global probe substrate for UGT enzymes (except for UGT1A4) based on the following. The kinetic behavior of 4-MU glucuronidation mediated by different UGT enzymes has been fully characterized^22^. Also, specific probe substrates for most individual UGTs have not been identified yet^23^ while 4-MU has been demonstrated to be a robust probe substrate in assessing the activity of most recombinant human UGTs in many studies^12^,^24^,^25^. Thus, characterization of UGT inhibition using 4-MU as a probe compound should provide solid preliminary data to estimate UGT inhibitory potentials of perpetrator drugs.

Important inhibition is uncommon for compounds with IC_50_ values greater than about 75–100 μM because sufficiently high levels are not clinically achieved^26^. So in the current study, for UGT isoforms whose activity is inhibited by >50% by individual TKIs at 100 μM, IC_50_ and *K*_i_ values of TKIs were further estimated. Most notable finding in our study was UGT inhibition by imatinib and lapatinib. Our study has revealed that imatinib is a broad inhibitor of several UGTs. Imatinib is a potent inhibitor of UGT2B17; an intermediate inhibitor of UGT1A1; and a weak inhibitor of UGT1A7, 1A8, 1A9, 1A10, 2B7, and 2B15. The human UGT2B enzymes are involved in the metabolism of steroid hormones as well as bile acids, acidic steroids, and fatty acids^27^. Among UGT2Bs, UGT2B17 exhibits high levels of activity against androgens and xenobiotics. UGT2B17 substrate drugs include carcinogens, coumarins, anthraquinones, flavonoids, nonsteroidal anti-inflammatory drugs, monoterpenoids, phenols, suberoylanilide hydroxamic acid (SAHA), and exemestane^28^,^29^,^30^. Therefore, the potent inhibition of UGT2B17 by imatinib could potentially have a significant clinical effect on the metabolism of UGT2B17 substrates. In fact, our results from quantitative prediction of DDI risk indicate that the co-administration of imatinib at clinical doses can result in over 2-fold increases in AUC of a UGT2B17 substrate drug.

Our data offer *in vitro* evidence that lapatinib is a potent inhibitor of UGT1A1. UGT1A1 is broadly expressed in human organs including liver, intestines, and kidney^31^,^32^,^33^; its expression levels in the intestines and kidney are one third as high as that in liver^11^. About 15% of top 200 prescribed drugs in the United States in 2002 are eliminated mainly via glucuronidation by UGT1A1^6^, and the inhibition of UGT1A1 can have clinically significant impacts on drug therapy with a narrow therapeutic index drug such as irinotecan. Irinotecan is a chemotherapeutic agent commonly used for the treatment of colorectal cancer. Irinotecan requires metabolic activation by carboxylesterase 2 to the active metabolite SN-38. SN-38 is mainly eliminated via glucuronidation by UGT1A1, with minor contribution by UGT1A3, 1A6, and 1A9^19^. Because UGT1A1 inhibition can exhibit substrate-dependency^18^, two UGT1A1 substrates, 4-MU and SN-38, were used in the kinetic study of lapatinib. Interestingly, the kinetic profile of inhibition of SN-38 glucuronidation was different from that of 4-MU glucuronidation. This finding offers new experimental evidence that the reduction of UGT1A1 activity might vary with the substrate, and also for the opinion that UGT1A1 has two or more binding sites for xenobiotics and endobiotics^18^. Our results from the quantitative prediction of DDI risk indicate that the coadministration of lapatinib at clinical doses could result in around 25% increase in AUC of SN-38. Because the extent of UGT inhibition in *in vivo* systems can be underestimated when the prediction is based solely on *in vitro* parameters^34^, our prediction of SN-38 and lapatinib interaction appears consistent with the clinical observation that co-administration of lapatinib increased the AUC of SN-38 by an average of 41%^16^.

Different from above two TKIs, vandetanib was found as an intermediate inhibitor of UGT1A9, which is also involved in the glucuronidation of a number of drugs^9^. UGT1A9 is expressed in both human liver and some extrahepatic tissues including the gastrointestinal tract^9^. Significant evidence exists supporting a role for gastrointestinal UGTs as modifiers of pharmacokinetics and biological responses to drugs and xenobiotics^35^. Our data show that vandetanib might influence the first-pass effect and bioavailability of more orally administered drugs. This issue requires additional attentions, especially when modern medicinal chemistry tends to synthesize polar chemicals to avoid the metabolism by CYPs, which makes Phase II enzymes become the main metabolizing enzymes such as UGTs.

It is noteworthy that the extrapolation from *in vitro* data to *in vivo* drug interactions should be taken with caution. Various factors may affect the accuracy of prediction, including enzyme resources, incubation systems, protein binding, active transporters, the selection of probe substrates, and the accumulation of products in the reaction medium so on. In addition, UGTs in the gastrointestinal tract may contribute significantly to the first-pass metabolism of orally administered drugs that undergo glucuronidation. The concentrations of substrates in the intestine may be different from the concentrations used here to predict the AUC ratio. Therefore, further systemic studies are needed to clarify the *in vivo* effects.

In summary, the present findings highlight potential DDIs involving specific TKIs and UGT substrates, namely by inhibition of UGT1A1 and 2B17 by lapatinib and imatinib, respectively. Our study provides a basis for design of clinical studies for investigation of DDIs involving TKIs.

## Methods

### Chemicals and Reagents

Axitinib, imatinib, lapatinib, vandetanib, SN-38 and SN-38 glucuronide (SN-38G) were purchased from Toronto Research Chemicals, Inc (Toronto, Ontario, Canada). Tris-HCl, 4-methylumbelliferone (4-MU), 4-methylumbelliferone-β-D-glucuronide (4-MUG), alamethicin and uridine 5-diphosphoglucuronic acid trisodium salt (UDPGA) were purchased from Sigma-Aldrich (St. Louis, MO). Trifluoperazine (TFP) was purchased from Enzo Life Science (Farmingdale, NY). All other reagents were of high-performance liquid chromatography (HPLC) grade or the highest grade commercially available. A panel of recombinant human UGT supersomes (UGT1A1, 1A3, 1A4, 1A6, 1A7, 1A8, 1A9, 1A10, 2B4, 2B7, 2B15, and 2B17) expressed in baculovirusinfected insect cells was purchased from BD Gentest (Woburn, MA). Pooled human liver microsomes (HLMs) (n = 50 donors) were purchased from Invitrogen (Carlsbad, CA).

### Inhibition of UGT Glucuronidation Assay

The nonselective substrate of UGTs, 4-MU, was used as a probe substrate for all UGT isoforms except for UGT1A4. A typical incubation mixture with a total volume of 100 μl contained recombinant UGT isoform (final concentration: 0.1, 0.15, 0.05, 0.01, 0.01, 0.01, 0.01, 0.5, 0.05, 0.15 and 0.1 mg/ml for recombinant UGT1A1, 1A3, 1A6, 1A7, 1A8, 1A9, 1A10, 2B4, 2B7, 2B15, and 2B17, respectively), 10 mM UDPGA, 5 mM MgCl_2_, 100 mM Tris-HCl buffer (pH 7.4), and 4-MU in the absence or presence of a TKI (100 μM). Incubations with 4-MU were performed at the final concentration corresponding to the apparent K_m_ or S_50_ value reported for each isoform (110, 1200, 110, 15, 750, 30, 80, 1200, 350, 250, and 2000 μM 4-MU for UGT1A1, 1A3, 1A6, 1A7, 1A8, 1A9, 1A10, 2B4, 2B7, 2B15, and 2B17, respectively)^12^. For UGT1A4, TFP was selected as the substrate. TFP (final concentration: 40 μM) was incubated with one of four TKIs and UGT1A4 (0.1 mg/ml). The following known UGT inhibitors were used as positive controls (at 100 μM): diclofenac for UGT1A1, 1A6, 1A7, and 1A9; hecogenin for UGT1A4, androsterone for UGT1A3, 2B7, and 2B15; and phenylbutazone for UGT1A8 and 1A10, respectively^12^,^22^,^34^. As there are no known inhibitors of UGT2B4 and 2B17, positive controls for these enzymes were not included. All the inhibitors and 4-MU or TFP were dissolved in DMSO. The final concentration of DMSO in the incubation system was 1% (v/v). The UGT reaction mixtures were pre-incubated at 37 °C for 5 min, and reactions were initiated by adding UDPGA (final concentration 5 mM). The following incubation times were used for different UGT isoforms: 120 min for UGT1A1, 1A10, 2B4, 2B7, 2B15, and 2B17; 75 min for UGT1A3; and 30 min for UGT1A4, UGT1A6, 1A7, 1A8, and 1A9. Rate of product formation for each isoform was linear with respect to protein concentration and incubation time. All the reactions were quenched by adding chilled acetonitrile (100 μl) containing phenytoin (1 μM) as an internal standard. The mixtures were centrifuged at 16,000 g for 16 min to obtain the supernatant. Samples were then analyzed in LC-MS/MS. The relative rate of 4-MUG or trifluoperazine glucuronide (TFPG) formation for each isoform was linear with respect to protein concentration and incubation time.

4-MUG concentrations were determined by using an Agilent 1200 HPLC interfaced with Applied Biosystems Qtrap 3200 equipped with an electrospray ion source. Chromatographic separation was carried out with a Waters XTerra MS C18 column (2.1 × 50 mm, 3.5 μm; Waters Corporation, Milford, MA). The mobile phase consisted of 0.1% formic acid in water (A) and acetonitrile (B) and was delivered at 250 μl/min. The gradient was initiated at 85% mobile phase A to 90% mobile phase B over 1 min, held constant for 1.5 min, and then restored to the initial composition until 10 min. The MRM parameters for 4-MUG and phenytoin as the internal standard were 353.05/177.20 and 253.30/182.20 in positive ion mode.

Signals for TFPG were detected by using Applied Biosystems Qtrap 4000 with an electrospray ion source interfaced with a Shimazu Prominence Modular HPLC. The mobile phase consisted of 0.1% formic acid in water (A) and acetonitrile (B). The column was first equilibrated at 25% mobile phase B for 1.5 min at a 200 μl/min flow rate. The elution was then ramped linearly to 90% mobile phase B over 1.5 min, maintained for 5 min, and followed by a return to initial conditions. TFPG was detected using MRM pair of 584/408 in positive ionization mode^36^. Phenytoin was used as the internal standard.

### Inhibition of SN-38 Glucuronidation Assay

SN-38 glucuronidation activity was determined with a slight modification of a previously published method^13^. SN-38 was incubated in the absence or presence of different concentrations of lapatinib (0–200 μM). Reactions were performed using recombinant UGT1A1 protein (0.1 mg/ml) or pooled HLMs (0.4 mg/ml). When HLMs were used, 50 μg/mg protein alamethicin was added into incubation systems for 15 min preincubation on ice. Alamethicin was dissolved in 90/10 incubation buffer/ethanol. The final concentration of ethanol was less than 0.1%. After preincubation for 5 min, the reaction was started by adding UDPGA (final concentration 5 mM). Incubation was performed at 37 °C for 30 min and stopped by adding chilled acetonitrile. The samples were processed as described above for analysis using LC-MS/MS.

The concentration of SN-38G was determined by using an Agilent 1200 HPLC interfaced with Applied Biosystems Qtrap 5500. The MRM setting for SN-38G was 569.2/393.2 in the positive mode. The same internal standard was used for quantification. The mobile phase consisted of 0.1% formic acid in water (A) and acetonitrile (B). The column was first equilibrated at 10% mobile phase B for 1 min at 250 μl/min. The elution was then ramped linearly to 90% mobile phase B over 2 min, maintained for 2 min, and followed by a return to initial conditions.

### Determination of IC_50_

The same incubation condition as for the preliminary inhibition study was used for each UGT isoform, except that the concentration of each inhibitor was ranged from 0 to 200 μM. The relative formation rate of 4-MUG, TFPG or SN-38G in the presence vs. absence of a TKI was estimated, and the half maximal inhibitory concentration (IC_50_) value for each TKI was estimated by using GraphPad Prism 5 software (La Jolla, CA).

### Determination of *K*_i_

4-MU at four concentrations (ranging from 0.5 × *K*_m_ to 4 × *K*_m_) was incubated with recombinant UGT1A1, 1A3, 1A7, 1A8, 1A9, 1A10, 2B7, 2B15 or 2B17 at a final protein concentration as described in the UGT glucuronidation inhibition assay in the presence or absence of TKI at four different concentrations (ranging from IC_50_/4 to 4 × IC_50_). The incubation time for each UGT isoform was the same as that used to determine IC_50_. SN-38 (2, 4, 10 or 20 μM) was incubated with UGT1A1 (0.1 mg/ml) or HLMs (0.4 mg/ml for) in the presence of lapatinib (0, 0.1, 0.5, 1 or 2 μM). *K*_i_ values were calculated using nonlinear regression according to the models for competitive inhibition, noncompetitive inhibition, or mixed inhibition. Global nonlinear regression analysis in the kinetics module of GraphPad Prism 5 software was used to simultaneously fit all data to the equations for competitive inhibition, non-competitive inhibition, and uncompetitive inhibition. The type of inhibition was determined based on Lineweaver-Burk plot and the best fitting of data to the equations. Goodness of fit to kinetic and inhibition models was assessed from the F statistic, *r*^2^ values, parameter standard error estimates and 95% confidence intervals. Kinetic constants are reported as the mean value ± standard error of the parameter estimate. Lineweaver-Burk plot and Dixon plot were also used as graphical representation of the type of inhibition and *K*_i_.

### Estimation of *in vivo* TKI Concentrations

The average systemic plasma concentration ([I]_av_), the maximum systemic plasma concentration ([I]_max_), the maximum unbound systemic plasma concentration ([I]_max,u_), the maximum hepatic input concentration ([I]_in_) and the maximum unbound hepatic input concentration ([I]_in,u_) of TKIs after repeated oral administration were estimated based on the previously reported equations^37^. The values of parameters needed were obtained from the literature^37^,^38^,^39^,^40^,^41^,^42^,^43^,^44^,^45^.

### Prediction of DDI Risk from *In Vitro* Data

The magnitude of the inhibition of TKIs was estimated as the ratio of the area under the curve (AUC_i_/AUC) of UGT substrates in the presence and absence of the inhibitor. The ratio was calculated based on the eq. 1 for drugs orally administered^37^:


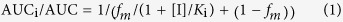


where AUC_i_ and AUC are the AUC in the presence and absence of inhibitor, respectively; *K*_i_ is inhibition constant from the *in vitro* inhibition experiment; *f*_m_ is the fraction of the UGT substrate metabolized by the inhibited enzyme; and [I] is the TKI concentration at the enzyme active site.

In view of the general assumption that only unbound drug is available for interaction with the enzyme active site, and the aim of DDI research is to prevent the highest potential risk, the maximum unbound hepatic input concentration ([I]_in,u_) was used for [I] in this study. Of note, clinically observed maximum unbound systemic plasma or blood concentration (C_max,u_) was not used because the C_max,u_ is known to be much lower than the [I]_in,u_^37^. Also, for the UGTs expressed in gastrointestinal track (such as UGT1A7), the maximum unbound systemic plasma concentration ([I]_max,u_) was used to represent [I]. The prediction of AUC ratio is for any potential UGT substrates with undetermined *f*_m_, a range of *f*_m_ between 0.1 and 1 was included for calculating AUC_i_/AUC ratio.

## Additional Information

**How to cite this article**: Zhang, N. *et al*. Drug-Drug Interaction Potentials of Tyrosine Kinase Inhibitors via Inhibition of UDP-Glucuronosyltransferases. *Sci. Rep*. **5**, 17778; doi: 10.1038/srep17778 (2015).

## Figures and Tables

**Figure 1:**
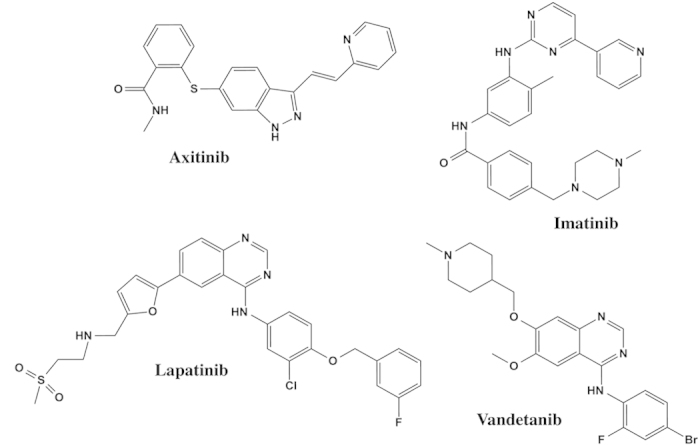
Chemical structures of axitinib, imatinib, lapatinib, and vandetanib.

**Table 1:** Remaining activities (%) of UGTs inhibited by 100 μM TKIs.

	Lapatinib	Vandetanib	Imatinib	Axitinib	Pos. Ctrl.
UGT1A1	30.8 ± 11.0	48.9 ± 4.9	34.1 ± 0.5	56.7 ± 3.4	37.0 ± 2.1
UGT1A3	96.5 ± 7.5	104.1 ± 2.1	88.8 ± 5.1	47.8 ± 12.8	39.4 ± 6.8
UGT1A4^a^	40.7 ± 1.9	62.8 ± 0.0	59.1 ± 3.0	14.9 ± 1.8	20.3 ± 6.6
UGT1A6	88.4 ± 5.9	55.1 ± 1.1	67.9 ± 7.1	85.8 ± 3.5	28.2 ± 10.0
UGT1A7	37.7 ± 9.3	22.7 ± 2.7	8.5 ± 3.1	25.0 ± 2.5	25.6 ± 11.2
UGT1A8	73.5 ± 10.6	56.0 ± 6.3	33.1 ± 9.0	54.6 ± 2.4	40.2 ± 5.3
UGT1A9	51.1 ± 7.9	14.1 ± 4.7	49.2 ± 1.9	44.8 ± 0.2	15.2 ± 1.2
UGT1A10	73.6 ± 4.8	49.0 ± 0.4	15.3 ± 9.5	55.5 ± 2.7	37.8 ± 8.0
UGT2B4	105.1 ± 0.2	80.8 ± 6.6	71.0 ± 3.2	42.1 ± 1.6	ND^b^
UGT2B7	90.5 ± 1.0	49.8 ± 3.7	29.8 ± 5.6	55.9 ± 9.7	16.4 ± 11.9
UBT2B15	109.5 ± 3.1	79.2 ± 1.4	28.3 ± 3.3	49.3 ± 5.2	21.6 ± 11.9
UBT2B17	94.8 ± 5.7	69.7 ± 3.1	14.7 ± 8.2	55.9 ± 0.3	ND

^a^TFP was used as a substrate, and for all other UGTs, 4-MU was used as substrate.

^b^no positive inhibitor available.

**Table 2:** The IC_50_ of TKIs for the inhibition of UGT activities.

	Axitinib (μM)	Imatinib (μM)	Lapatinib (μM)	Vandetanib (μM)
UGT1A1	ND^a^	11.0 ± 2.8	0.5 ± 0.0	ND
UGT1A3	ND	ND	ND	ND
UGT1A4^b^	14.2 ± 3.2	ND	3.8 ± 0.7	ND
UGT1A7	3.3 ± 0.7	19.5 ± 4.1	2.7 ± 1.4	38.9 ± 4.3
UGT1A8	ND	22.9 ± 3.8	ND	ND
UGT1A9	3.6 ± 0.7	ND	ND	5.0 ± 0.4
UGT1A10	ND	47.3 ± 7.6	ND	ND
UGT2B7	ND	68.4 ± 10.8	ND	ND
UGT2B15	ND	91.0 ± 18.3	ND	ND
UGT2B17	ND	0.8 ± 0.1	ND	ND

^a^not determined; based on results from preliminary experiments showing that the extent of UGT inhibition at 100 μM of a respective TKI was <50%.

^b^TFP was used as a substrate.

**Table 3:** The inhibition kinetic parameters and types of TKIs on UGTs.

enzymesresource	Substrate	*K*_i_ (μM)/inhibition type		
		Imatinib	Lapatinib	Vandetanib
UGT1A1	4-MU	19.1 ± 2.8/competitive	0.5 ± 0.1/competitive	–
UGT1A1	SN-38	–	0.6 ± 0.1/competitive	–
UGT1A4	TFP	–	36.4 ± 5.0/mixed	–
UGT1A7	4-MU	–	4.5 ± 0.5/noncompetitive	–
UGT1A9	4-MU	–	–	9.0 ± 1.2/mixed
UGT2B17	4-MU	0.4 ± 0.1/competitive	–	–
HLMs	SN-38	–	1.6 ± 0.2/noncompetitive	–

**Table 4:** Pharmacokinetic parameters of TKIs used in the estimation of AUC changes.

Drugs		Axitinib	Imatinib	Lapatinib	Vandetanib
Dose (mg)		5	400	1250	300
Dosing Interval (hr)		12	24	24	24
C_max_ (μM)		0.16	4.41	4.2	3.32
Absorption Rate Constant (k_a_, h^-1^)		0.523	1.64	0.7	0.3
Fraction absorbed (F_a_)		0.88	1	0.73	0.935
Plasma Unbound Fraction (f_u,p_)		0.01	0.05	0.01	0.06
Oral CL (L/h)		45	11.2	114	13.2
t_1/2_ (h)		3.2	22	24	456
Calculated Concentrations	I_av_ (μM)	0.024	2.52	0.79	1.99
	I_max,u_ (μM)	0.00067	0.18	0.011	0.12
	I_in,u_ (μM)	0.00086	0.7	0.12	0.23
References		17,40,45	38,39,46	41,42	43,44
